# Crash Diagnosis and Price Rebound Prediction in NYSE Composite Index Based on Visibility Graph and Time-Evolving Stock Correlation Network

**DOI:** 10.3390/e23121612

**Published:** 2021-11-30

**Authors:** Yuxuan Xiu, Guanying Wang, Wai Kin Victor Chan

**Affiliations:** 1 Tsinghua Shenzhen International Graduate School, Tsinghua University, Shenzhen 518055, China; yuxuanxiu@gmail.com; 2 Tsinghua-Berkeley Shenzhen Institute, Tsinghua University, Shenzhen 518055, China; 3 College of Management and Economics, Tianjin University, Tianjin 300072, China; wangguanyingnk@163.com

**Keywords:** stock market, crash, rebound, log-periodic power law, visibility graph, stock correlation network, anomaly detection, extreme value theory

## Abstract

This study proposes a framework to diagnose stock market crashes and predict the subsequent price rebounds. Based on the observation of anomalous changes in stock correlation networks during market crashes, we extend the log-periodic power-law model with a metric that is proposed to measure network anomalies. To calculate this metric, we design a prediction-guided anomaly detection algorithm based on the extreme value theory. Finally, we proposed a hybrid indicator to predict price rebounds of the stock index by combining the network anomaly metric and the visibility graph-based log-periodic power-law model. Experiments are conducted based on the New York Stock Exchange Composite Index from 4 January 1991 to 7 May 2021. It is shown that our proposed method outperforms the benchmark log-periodic power-law model on detecting the 12 major crashes and predicting the subsequent price rebounds by reducing the false alarm rate. This study sheds light on combining stock network analysis and financial time series modeling and highlights that anomalous changes of a stock network can be important criteria for detecting crashes and predicting recoveries of the stock market.

## 1. Introduction

A stock market crash is one of the most significant systemic risks of the modern financial system, causing significant losses for investors. A recent example is the March 2020 stock market crash triggered by COVID-19 [1], during which the New York Stock Exchange (NYSE) Composite Index plunged roughly 35% within a month. Meanwhile, rebounds of the stock market after crashes usually signal the recovery of investors’ confidence or the taking effect of bailout policies. Therefore, it is critical for investors and policy makers to detect stock market crashes and predict price rebounds.

In the past decades, different methods have been proposed to diagnose the stock market crashes and predict rebounds. One of the most representative methods is the log-periodic power-law (LPPL) model [2]. Originally, the LPPL model was proposed to predict the bursting point of financial bubbles. Yan et al. [3] adopt the LPPL model to study stock market crashes by considering them as the “mirror images” of financial bubbles, which are also known as “negative bubbles”. The fundamental insight of modeling financial crashes with the LPPL model is to capture a particular pattern of the price time series, which can be described as “the faster-than-exponential decline accompanied by accelerating oscillations” [4]. The LPPL model and its extensions are successfully applied in diagnosing negative bubbles of many types of assets, such as crude oil [5] and cryptocurrency [6].

Despite previous achievements, all the existing LPPL-based models only focus on the price time series itself. However, it may not be sufficient to use only the price time series of the stock index when diagnosing stock market crashes. The price time series of the stock index can describe the overall fluctuation of the stock market, but it ignores the complex interactions among multiple assets.

Network analysis provides a novel tool for characterizing the complex interactions and co-movements in the stock market [7,8,9]. In fact, it has been discovered that stock market crashes and recoveries are accompanied by drastic changes in the topological structure of stock correlation networks, which can be captured by some network statistics such as assortativity [10], modularity [11], von Neumann entropy [12], the structural entropy [13,14] and the graph motif entropy [15]. Although the above literature qualitatively analyzes the dynamical changes of stock correlation networks during stock market crashes and rebounds, there is still a lack of work that applies such phenomenon to quantitatively diagnose stock market crashes and predict price rebounds.

This paper contributes to the literature by incorporating the analysis of a time-evolving stock correlation network into the LPPL model. The contribution of this paper has three folds. First, extending the LPPL model, we propose to characterize stock market crashes by two distinct characteristics: (1) faster-than-exponential decline of the stock index price; (2) abnormal changes in the market structure (i.e., the topology of the stock correlation network). Second, we design a prediction-guided anomaly detection method based on the extreme value theory (EVT) in order to define and detect “anomalies” for Characteristic (2). The intuition is that a properly trained predictor can forecast most of the “normal” situations well, with significant deviations when “abnormal” situations occur. The “normal” and “abnormal” deviations are distinguished based on EVT. Third, we propose a framework by combining Characteristic (1) and (2), where Characteristic (1) is captured by a visibility graph (VG)-based representation of the LPPL model proposed by Yan et al. [16]. A hybrid rebound indicator is calculated, which is a linear combination of Yan’s VG-based indicator and the anomalies of the stock correlation network. Specifically, we normalize the anomalies to 0 to 1 based on EVT and treat them as confidence levels (i.e., weights) of the VG-based rebound indicator.

Experiments are conducted based on the data of the New York Stock Exchange (NYSE) Composite Index. We predict the subsequent price rebounds of the 12 major crashes in the U.S. stock market from 4 January 1991 to 7 May 2021. Experimental results demonstrate that our proposed prediction-guided anomaly detection algorithm is well capable of identifying abnormal changes in stock correlation networks during market crashes and recoveries. Furthermore, our proposed hybrid indicator outperforms Yan’s VG-based indicator by reducing the false alarm rate. These findings imply that incorporating the analysis of the time-evolving stock correlation network into the modeling of the stock index time series is a promising direction for diagnosing and predicting financial markets.

The rest of this paper is organized as follows. Section 2 describes the data. Section 3 introduces our proposed framework for stock market crash diagnosis and rebound prediction. Section 4 presents the experimental results. Section 5 discusses the main findings, implications and limitations of this study. Section 6 concludes our work and provides future research directions.

## 2. Data Description and Labeling

### 2.1. Data Description

This paper uses the daily closing price of the NYSE Composite Index, which is collected from the Yahoo! financial database (http://finance.yahoo.com) (accessed on 25 November 2021). The time period is from 2 January 1986 to 7 May 2021, with the data from 2 January 1986 to 3 January 1994 as the training set and the rest of the data as the testing set. As shown in Table 1, there are 15 major crashes in the U.S. stock market, including 3 in the training set and 12 in the testing set. The list of stock market crashes in the U.S. before 2008 is provided in [15], while the crashes after 2008 are manually collected from Wikipedia (https://en.wikipedia.org/w/index.php?title=List_of_stock_market_crashes_and_bear_markets) (accessed on 25 November 2021). Furthermore, we select 199 stocks out of the 347 stocks in the NYSE dataset [11], whose data are available for the entire time period from 1986 to 2021. Their price time series are used to construct the time-evolving stock correlation network.

### 2.2. Labeling Rebounds of Price Time Series

The rebounds of the financial market are labeled based on the price time series of the stock index. Following the definition in the existing literature [16,17], we define the rebound as the time point at which a stock index turns from a downtrend to an uptrend after a stock market crash. This paper labels the trend of the stock index based on a recently proposed method [18]. The basic idea is that the price time series is considered to change from an upward trend to a downward trend when the price falls by more than *w* compared with the local peak. Similarly, when the price rises above *w* compared with the local trough, the price time series is considered to change from a downward trend to an upward trend. Here, *w* is a predetermined threshold for the proportion of price increases and decreases. The detailed procedure is illustrated in Appendix A.

**Figure 1:**
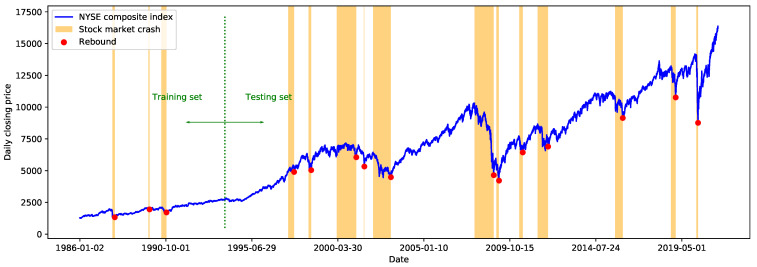
The NYSE Composite Index with labeled crashes and rebounds.

For each stock market crash in Table 1, we detect the first turning point when the trend of the price time series changes from a downtrend to an uptrend. This turning point is defined as the time point of the price rebound after the corresponding stock market crashes. Here we choose w=0.15, which is an empirical value proposed along with the trend labeling method [18]. In other words, once the price rises by more than 15% from the local trough after a financial crash, it is regarded as a switch from a downtrend to an uptrend, and the local trough is labeled as a rebound. The labeling results are further manually adjusted according to the historical records. Figure 1 shows the time series of the daily closing price of the NYSE Composite Index, as well as the labeled price rebounds and the corresponding stock market crashes.

## 3. Methodology

Our proposed framework predicts market rebounds by observing and quantifying a specific phenomenon, which is a faster-than-exponential decline in the stock index price time series accompanied by anomalous changes in the topology of the correlation network of constituent stocks. Therefore, our proposed framework consists of the following four steps: (1) quantifying faster-than-exponential decline in stock index price; (2) constructing time-evolving stock correlation network; (3) detecting anomalous topological changes of stock correlation networks; (4) calculating a rebound alarm index. In this section, we first introduce our proposed framework and then describe each of these four steps.

### 3.1. Our Proposed Framework

**Figure 2:**
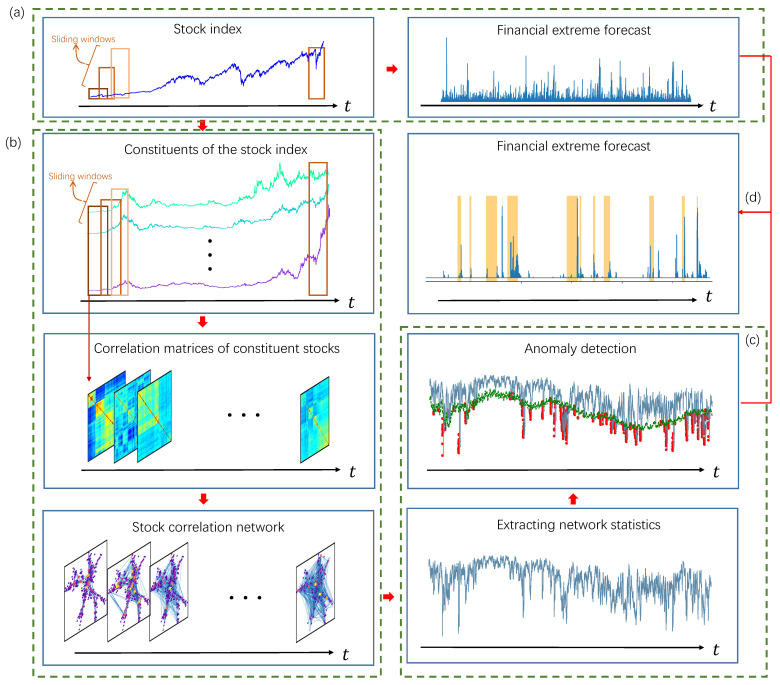
Our proposed framework.

Our proposed framework is illustrated in detail in Figure 2. We first measure the faster-than-exponential decline of the price time series of the stock index based on Yan’s VG-based LPPL model [16] (step *a*). The detailed procedures are described in Section 3.2.

The time series of the stock index can describe the overall fluctuation of the financial market, but it ignores the complex interactions between different assets. In our proposed framework, we exploit information of the market structure by investigating the time-evolving topology of the stock correlation network (step *b* and *c*). In step *b*, we first select the constituents of the stock index, then calculate the matrices of Pearson correlation coefficients based on the logarithmic return of the constituents through a sliding window, and finally extract the most important correlations in the matrix to form a stock correlation network. In step *c*, topological measurements are extracted from the time-evolving stock correlation network. Anomaly detection is performed on the extracted topological measurements to identify the emergence and disappearance of anomalous network topology. Section 3.3–Section 3.5 describe the implementation of step *b*, *c* and step *d*, respectively.

### 3.2. Quantifying Faster-Than-Exponential Decline in Stock Index Price

We adopt the method based on the visibility graph [16] to quantitatively describe the faster-than-exponential decline of the stock index prices. This subsection first briefly introduces the basic idea of the visibility graph and then describes the quantification of the faster-than-exponential decline of the stock index price based on the VG.

The visibility graph (VG) is first presented by Lacasa et al. [19] as an algorithm for converting time series into complex networks. Its basic idea is to map each time point in a discrete time series into a node in a network. The edges of the network are established based on the visibility criteria. The detailed procedure of constructing the visibility graph is introduced as follows.

For each pair of data points in the time series noted as $\left(t_{i},y_{i}\right)$ and $\left(t_{j},y_{j}\right)$ where i<j, the VG algorithm first connects them with a straight line. If all the data points between *i* and *j* are below the straight line, $\left(t_{i},y_{i}\right)$ and $\left(t_{j},y_{j}\right)$ are considered “visible” to each other, and an edge is created between them in the visibility graph. A more formal expression is: for a time series $\left[\left(t_{1},y_{1}\right),\left(t_{2},y_{2}\right),\cdots ,\left(t_{N},y_{N}\right)\right]$ of length *N*, the adjacency matrix *W* of its visibility graph is defined as  

(1) $$\omega_{ij}=\left\{\begin{array}{cc} 1, & \;\mathrm{if}\;y_{k}<y_{i}+\frac{t_{k}-t_{i}}{t_{j}-t_{i}}\left(y_{j}-y_{i}\right),\forall i<k<j\\ 0, & \;\mathrm{otherwise}\; \end{array}\right.,$$

where $\omega_{ij}$ denotes the weight of the edge (i,j) in the visibility graph.

Yan et al. [16] first represent the log periodic power law (LPPL) model with the visibility graph. Originally, the LPPL model characterizes the financial bubbles by the faster-than-exponential growth of the stock market prices. Yan et al. construct the visibility graph based on the logarithmic prices of stock indices. Since the exponential growth is a straight line in the logarithmic-linear scale, faster-than-exponential growth is represented as a convex curve. Therefore, as the price time series goes up super-exponentially, the degree of the last node of its VG increases. Furthermore, if two nodes $\left(t_{i},y_{i}\right)$ and $\left(t_{j},y_{j}\right)$ are “visible” to each other, the growth rate from $t_{i}$ to $t_{j}$ can be roughly considered as “faster-than-exponential”.

Since our goal is to characterize the financial crashes, we adopt the absolute invisibility graph, which is exactly the opposite of the visibility graph. Its adjacency matrix $W={\left\{\omega_{ij}\right\}}_{i,j=1,\cdots ,N}$ is defined as

(2) $$\omega_{ij}=\left\{\begin{array}{cc} 1, & \;\mathrm{if}\;y_{k}>y_{i}+\frac{t_{k}-t_{i}}{t_{j}-t_{i}}\left(y_{j}-y_{i}\right),\forall i<k<j\\ 0, & \;\mathrm{otherwise}\; \end{array}\right..$$

Similar to the visibility graph, if two nodes $\left(t_{i},y_{i}\right)$ and $\left(t_{j},y_{j}\right)$ are “absolutely invisible” to each other, we can roughly conclude a faster-than-exponential decline from $t_{i}$ to $t_{j}$. For each data point $\left(t_{i},y_{i}\right)$ in a time series, we obtain $T_{\mathrm{VG}}-1$ historical data before $t_{i}$ through a sliding window of length $T_{\mathrm{VG}}$ to construct the absolute invisibility graph. The magnitude of the faster-than-exponential decline at time point $\left(t_{i},y_{i}\right)$ is defined as

(3) $$I_{\mathrm{VG}}\left(i\right)=\frac{1}{T_{\mathrm{VG}}}\sum_{j=i-T_{\mathrm{VG}}}^{i-1}1_{\left[y_{i}<y_{j}\right]}\cdot 1_{\left[y_{k}>y_{i}+\frac{t_{k}-t_{i}}{t_{j}-t_{i}}\left(y_{j}-y_{i}\right)\right]},$$

where $1_{\left[\cdot \right]}$ is the indicator function.

### 3.3. Constructing the Time-Evolving Stock Correlation Network

This paper studies the time-evolving nature of the correlation network, which is formed by the constituents of a stock index. Our basic assumption is that the topology of the stock correlation network changes significantly during financial crashes, and that the recovery of the network topology implies the recovery of the financial market.

The most straightforward way of studying a time-evolving network is to look at snapshots of the network taken at different time points. By stacking the snapshots in temporal order, a time-evolving network is denoted as $G=\left(G_{1},\ldots ,G_{T}\right)$. Each $G_{t}=\left\{N_{t},E_{t}\right\}$ is a snapshot recorded as time *t*, where $N_{t}$ is the set of nodes and $E_{t}$ is the set of edges. Since this paper considers the time-evolving correlations among the same set of stocks, all the snapshots in G share the same set of nodes, that is, $N_{1}=N_{2}=\cdots =N_{T}=N$.

In this paper, each snapshot $G^{t}$ is obtained from the correlation matrix $C^{t}$ by a filtering method. The construction procedure is composed of four steps: (1) dividing time windows; (2) determining the constituents of the stock index; (3) calculating correlation matrices; (4) extracting single layer networks from the correlation matrices. The detailed procedure is illustrated as follows.

#### 3.3.1. Dividing Time Windows

In terms of dividing time windows, three parameters need to be determined: (1) the length $T_{\mathrm{tw}}$ of the time window, (2) the step size Δ between two consecutive windows. The choice of time window length $T_{\mathrm{tw}}$ is a trade-off between over-smoothed and too noisy data [20]. In order to capture the dynamics of stock market correlations, we need to choose the smallest possible window length. On the other hand, the time window needs to be long enough to avoid the Epps effect [21]. Here, we choose a commonly used empirical value $T_{\mathrm{tw}}=25$ [15], meaning that each time window contains 25 trading days. To have a continuous tracking of the stock correlations, the step size is set as Δ=1, shifting the time window 1 day forward at each step.

#### 3.3.2. Determining Constituents of the Stock Index

In this step, we determine the name list of the stocks to track, that is, the set of nodes N. Notice that the constituents of the stock index are constantly adjusted over time. To ensure continuous and stable tracking of the market structure, we select the constituents of the stock index whose historical data are available during the entire experimental period. In this paper, we select N=199 stocks from the NYSE dataset [11,22], whose daily closing prices are available from 2 January 1986 to 7 May 2021.

#### 3.3.3. Calculating Correlation Matrices

For stock *i* in $M^{t}$, its logarithm return at time *t* is defined as

(4) $$r_{i}\left(t\right)=\ln p_{i}\left(t\right)-\ln p_{i}\left(t-1\right),$$

where $p_{i}\left(t\right)$ is the adjusted closure price of stock *i* at time *t*. Then the Pearson correlation coefficient between stock *i* and stock *j* is calculated as

(5) $$c_{ij}\left(t\right)=\frac{E\left[r_{i}\left(t\right)⊙r_{j}\left(t\right)\right]-E\left[r_{i}\left(t\right)\right]E\left[r_{j}\left(t\right)\right]}{\sqrt{\left(E\left[r_{i}^{2}\left(t\right)\right]-{\left(E\left[r_{i}\left(t\right)\right]\right)}^{2}\right)\left(E\left[r_{j}^{2}\left(t\right)\right]-{\left(E\left[r_{j}\left(t\right)\right]\right)}^{2}\right)}},$$

where E(·) represents the sample mean, ⊙ denotes element-wise multiplication of vectors and

(6) $$r_{i}\left(t\right)=\left[r_{i}\left(t-T_{\mathrm{tw}}+1\right),r_{i}\left(t-T_{\mathrm{tw}}+2\right),\cdots ,r_{i}\left(t\right)\right]$$

is the logarithm return series of stock *i* within the time window. Thus a N×N correlation matrix C(t) is obtained.

#### 3.3.4. Extracting Single-Layer Networks from Correlation Matrices

The threshold-based method [23] is applied to extract the strong correlations and construct the network. For the correlation matrix C(t), we choose a threshold ρ(t) and only keep $c_{ij}\left(t\right)>\rho \left(t\right)$ as the edges of the network $G^{t}$. This paper sets ρ(t) as the 85th percentile of all elements in C(t). The connection criterion of network $G^{t}$ is formally defined as

(7) $$g_{ij}^{t}=\left\{\begin{array}{cc} 1, & \;\mathrm{if}\;c_{ij}\left(t\right)>\rho \left(t\right)\\ 0, & \;\mathrm{otherwise}\; \end{array}\right.,$$

where $g_{ij}^{t}$ denotes the weight of the edge (i,j) in the network $G^{t}$.

#### 3.3.5. Calculating Singular Value Decomposition Entropy

We further measure the topology of each layer $M^{t}$ by the singular value decomposition (SVD) entropy, which has been applied to the analysis of financial market networks [24,25,26,27]. The definition of the SVD entropy is introduced below. It is worth noting that the topological characteristic is not limited to the SVD entropy, any proper network statistic, such as the von Neumann entropy [28] or the graph motif entropy [15], is applicable to our proposed framework.

The SVD entropy is based on the singular value decomposition of the N×N adjacent matrix *A* of the network *G*,

(8) $$A=U\Sigma V^{T},$$

where Σ is a diagonal matrix of singular values,

(9) $$\Sigma =\mathrm{diag}\left(\sigma_{1},\cdots ,\sigma_{N}\right),$$

The SVD entropy is defined as

(10) $${\mathrm{Ent}}_{t}=-\sum_{i}{\bar{\sigma }}_{i}\ln\left({\bar{\sigma }}_{i}\right),$$

where ${\bar{\sigma }}_{i}$ is the normalized singular value defined as

(11) $${\bar{\sigma }}_{i}=\frac{\sigma_{i}}{\sum_{j}\sigma_{j}}.$$

By calculating the topological characteristics, we transform the time-evolving network into a time series, which is much easier to interpret. Based on the time series of the SVD entropy, we identify and measure anomalous changes in the topology of the stock temporal network in the following subsections.

### 3.4. Prediction-Guided Anomaly Detection Based on Extreme Value Theory

In this subsection, we detect the anomalous value of each of the topological indicators based on the extreme value theory (EVT) and time series prediction. Our intuition is that we first train advanced time series forecasting algorithms only based on “normal” values. We assume that such predictors are able to capture the “normal” dynamics properly, while being completely unaware of abnormal changes in the dynamics. Based on the commonly adopted normal distribution assumption of the forecasting error, the residuals between the predicted and true value should be small and normally distributed for “normal” data, while the residuals of “abnormal” data should be large and their distribution can be portrayed by EVT. We determine the threshold between the “normal” and “abnormal” residuals based on the training dataset, as well as the parameters of the distribution of the extreme values.

Subsequently, we make predictions and calculate the residual for each day in the testing dataset. If the residual exceeds the threshold, we treat it as an “abnormal” value and calculate its “anomaly score” based on the extreme value distribution. Inspired by [29], our designed method can be divided into an initialization step and an execution step, whose detailed procedures are described below.

#### Initialization Step

In the initialization step, we first generate the training set that only contains “normal” data. The detailed procedure is described in Appendix B. The training set is generated to train a predictor F(·). Without loss of generality, we assume that the predictor makes single-step predictions based on data from the previous *d* days as

(12) $$I_{t}=F\left(I_{t-d},\cdots ,I_{t-1}\right)+\epsilon \left(t\right),$$

where ϵ(t) is a normally distributed error term.

To ensure that the predictor only learns the “normal” dynamics of the time series, we generate a training set that only contains the “normal” values. We first split time series I into different segments based on the date of the financial crashes. For example, the first segment is the “normal period” from 1 January 1986 to 15 October 1987, which is followed by the financial crash from 19 October 1987 to 4 December 1987. We then extract training samples from the “normal periods” using a sliding window of length d+1, where the data of the first *d* days are the inputs to the predictor, and the last datum is the expected output. Finally, we obtain the set of the training inputs $S_{x}$ and its corresponding target output set $S_{y}$ based on which the predictor F(·) is trained.

After training the predictor, for each day *t* in the first *N* days, we make the prediction based on the previous *d* days as

(13) $${\hat{I}}_{t}=F\left(I_{t-d},\cdots ,I_{t-1}\right).$$

Since we are looking for extremely small values, the residual is calculated as $X_{t}={\hat{I}}_{t}-I_{t}$. Notice that here we consider both the “normal” periods and the financial crashes, so the set of the residuals should contain the extreme values corresponding to the financial crashes. Therefore, we analyze the tail distribution of the residual based on the EVT.

According to the EVT, the extreme values always follow the same type of distribution, regardless of the initial distribution of the data. It can be regarded as a theorem for the maximum values, which is similar to the central limit theorem for the mean values [30]. A mathematical formulation is provided by the Pickands–Balkema–de Haan theorem [31,32], which can be written as:

(14) $${\bar{F}}_{t}\left(x\right)=P\left(X-\tau >x|X>\tau \right)∼{\left(1+\frac{\xi x}{\sigma }\right)}^{-\frac{1}{\xi }}.$$

This theorem shows that, for a random variable $X_{t}$, the excess over a sufficiently large threshold τ tends to follow a generalized Pareto distribution (GPD) with parameters ξ and σ [29].

A practical implication of EVT is that extreme and non-extreme events follow different distributions because they are often generated by different driving forces [33]. This is the theoretical basis for our use of EVT to identify abnormal changes in network topology. We argue that normal and abnormal residuals are caused by different driving forces, thus we aim to find the outliers that follow GPD. Based on the idea of EVT, we select the most appropriate threshold τ that allows GPD to fit the distribution of X−τ properly. This means that any value greater than τ can be regarded as an extreme value. Therefore, we consider a residual above the threshold τ as an anomalous value. The detailed procedure for obtaining the optimal value of τ is described in Appendix C.

### 3.5. Execution Step and Hybrid Rebound Indicator

The detailed procedure of the execution step is shown in Algorithm A4. It is designed to deal with the streaming data. For each day *t*, we calculate the residual $X_{t}=F\left(I_{t-d},\cdots ,I_{t-1}\right)-I_{t}$. If the residual exceeds the threshold τ, we raise an alarm while calculating the corresponding alarm index by

(15) $$I_{\mathrm{Alm}}\left(t\right)=\mathrm{Alm}\left(X_{t}\right)=1-{\left[1+\frac{\xi (X_{t}-\tau )}{\sigma }\right]}^{-\frac{1}{\xi }}.$$

Notice that our alarm index is actually the CDF of the generalized Pareto distribution $F_{\xi ,\sigma }\left(X_{t}-\tau \right)$.

Considering the development of the financial market, the internal dynamics of the topological changes of the stock correlation network may also be changing. Therefore, we need to constantly update the predictor based on the new data. Because the predictor is not expected to learn any information about the abnormal changes of the network, we only include the “normal” data into the training set. After every *K* new samples are added into the training set, the predictor is retrained to ensure that it keeps tracking the latest dynamics of the system.

We finally propose an indicator to characterize the phenomenon of “faster-than-exponential decline in the stock index price accompanied by anomalous changes in the market structure”. The alarm index defined in Equation (15) has a value range of (0,1), and its magnitude indicates the extent to which we believe the network structure is anomalous. Therefore, it can be considered as a “confidence level”, which is used as a mask to multiply the indicator defined in Equation (3). Considering that the anomalous changes in the stock correlation network are not necessarily perfectly synchronized with the plunge in the stock index, we make a moving average smoothing of $I_{\mathrm{Alm}}\left(t\right)$ with sliding window length $T_{\mathrm{Alm}}$. Notice that $T_{\mathrm{Alm}}$ should be a small integer. Based on the intuition that information from two weeks ago is hardly useful for forecasting, here we take $T_{\mathrm{Alm}}<10$. Since our proposed rebound indicator considers both the time series and the network, it is named as a hybrid indicator, whose formal definition is given as

(16) $$I_{\mathrm{Hybrid}}\left(t\right)=I_{\mathrm{VG}}\left(t\right)\cdot \left(\frac{1}{T_{\mathrm{Alm}}}\sum_{i=0}^{T_{\mathrm{Alm}}-1}I_{\mathrm{Alm}}\left(t-i\right)\right).$$

## 4. Experimental Results

In this section, we first qualitatively compare our proposed hybrid indicator with the baseline method [16] to give a general idea of how the analysis of the time-evolving stock correlation network helps to improve the prediction performance. We further quantitatively analyze the predictive power of our proposed framework based on the commonly adopted error diagram method. Experimental results demonstrate the effectiveness and robustness of our proposed method.

### 4.1. Qualitative Observation

In this subsection, we construct three indicators: (1) the VG-based indicator $I_{\mathrm{VG}}\left(t\right)$; (2) the alarm index $I_{\mathrm{Alm}}\left(t\right)$; (3) the hybrid indicator $I_{Hybrid}\left(t\right)$. The look-back scope of $I_{\mathrm{VG}}\left(t\right)$ is set as $T_{\mathrm{VG}}=262$, which is the same as the original paper [16]. The prediction algorithm for calculating $I_{\mathrm{Alm}}\left(t\right)$ is the Prophet forecasting model [34], whose open-source implementation is available at https://github.com/facebook/prophet (accessed on 25 November 2021). We use the data from 2 January 1986 to 3 January 1994 as the training set, and use the rest of the data as the testing set. The free parameter $T_{\mathrm{Alm}}$ is set as $T_{\mathrm{Alm}}=4$.

**Figure 3:**
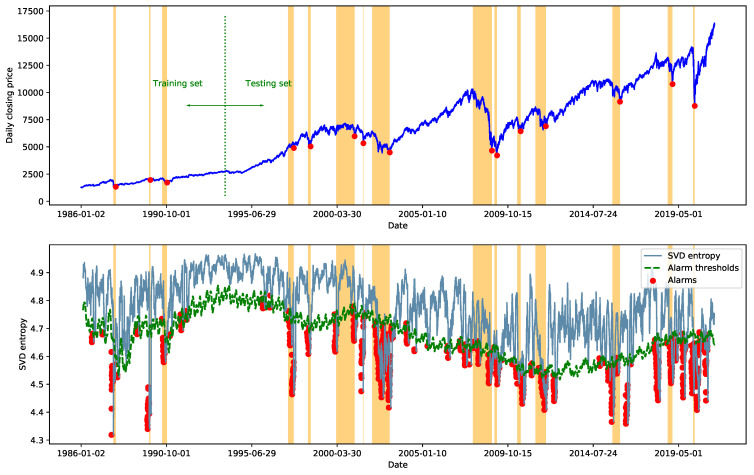
Result of the prediction-guided anomaly detection procedure.

Figure 3 demonstrates the result of the prediction-guided anomaly detection procedure. The solid blue line in the figure indicates the SVD entropy of the stock correlation network for each day. The green dashed line indicates the alarm thresholds obtained based on the one-day ahead prediction, and each red dot indicates that an alarm is issued on that day. An alarm for the financial crisis will be raised on day *t*, if the SVD entropy on day *t* falls below the predicted alarm threshold on that day.

**Figure 4:**
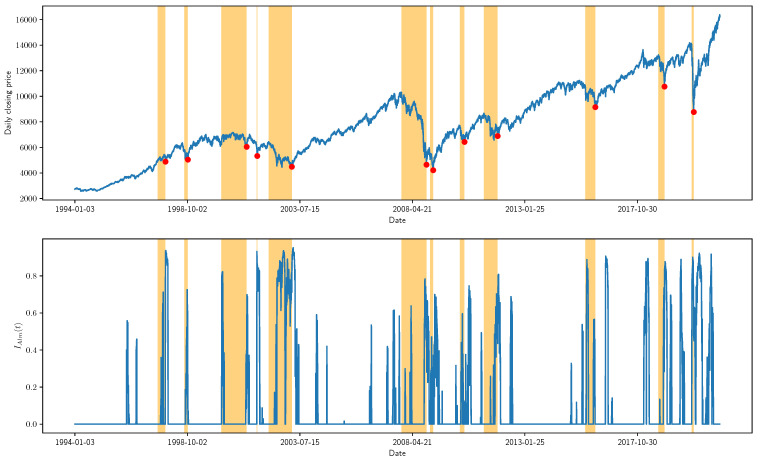

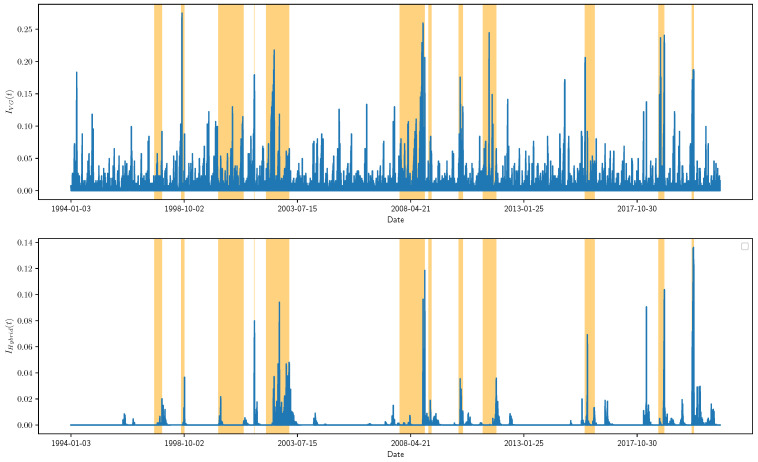
The NYSE Composite Index with three indicators.

Figure 4 shows the price time series of the NYSE Composite Index as well as the three indicators. For the alarm index $I_{\mathrm{Alm}}\left(t\right)\in \left(0,1\right)$, a higher value indicates the higher confidence that the network structure on day *t* is anomalous. Thus, we can see that most of the financial crashes are accompanied by an anomalous change in the topology of the stock correlation network. However, an abnormal network topology does not necessarily imply the occurrence of a financial crisis. Similarly, a high $I_{\mathrm{Alm}}\left(t\right)$ usually implies that the stock index is close to a local trough, but there also exists a large number of false alarms. Our approach effectively suppresses the false alarms by considering both the faster-than-exponential decline in the stock index (i.e., $I_{\mathrm{VG}}$) and the anomalous changes in the topology of the stock correlation network (i.e., $I_{\mathrm{Alm}}$).

### 4.2. Error Diagram

In this subsection, the predictive power of our proposed framework is evaluated based on the widely-adopted error diagram [4,16]. We also compare the short-term and long-term prediction performance of our proposed framework with the VG-based baseline method.

Error diagrams are often used to decide whether an indicator has predictive power for events that are difficult to predict, such as earthquakes [35,36,37] and financial extremes [3,16,38]. Just like the ROC (receiver operating characteristic) curve, the error diagram demonstrates the prediction performance of a certain indicator under different determination criteria (e.g., thresholds). Its x-axis is the “alarm ratio” $R_{\mathrm{Alarm}}$, which is defined as the total number of alarms divided by the length of the testing period. The y-axis is the ratio of missed events $R_{\mathrm{Miss}}$, which is defined as the number of missed events divided by the total number of events in the testing period. Therefore, the prediction performance of a random guess is represented by the straight line y=1−x in the error diagram. Any prediction method that is better than a random guess follows a curve below the anti-diagonal y=1−x. Furthermore, the *p*-value of the hypothesis that the prediction indicator is better than a random guess is $p=A/A_{unit}=A$, where *A* is the area under a curve (AUC) of the error diagram and $A_{unit}=1$ [16].

The error diagram of predicting financial rebounds of a stock index is created in the following way [4]:

1. Determine the forward-looking period *a*, that is, if a rebound occurs within *a* days after an alarm being raised, we consider the rebound has successfully predicted the alarm.
2. Count the number of rebounds in the testing set according to the definition in Section 2.
3. Sort the values of the rebound indicator time series in a decreasing order and save them in $I_{sort}$. The largest value in $I_{sort}$ is the first threshold.
4. We check the rebound indicator of each day during the testing period. If the rebound indicator on day *t* exceeds the predetermined threshold, an alarm is raised. If a rebound occurs during day *t* to day t+a, we consider the alarm successfully predicts the rebound.
5. We compare the successful predictions of the current threshold and the previous threshold. If there is no new successful prediction, the threshold is moved down to the next value in $I_{sort}$.
6. If new predictions are made based on a threshold, we count the missed rebounds and calculate the ratio of missed events as
  (17) $$R_{\mathrm{Miss}}=\frac{\mathrm{Number}\;\mathrm{of}\;\mathrm{missed}\;\mathrm{rebounds}}{\mathrm{Total}\;\mathrm{number}\;\mathrm{of}\;\mathrm{rebounds}}$$

The alarm ratio is calculated as

(18) $$R_{\mathrm{Alarm}}=\frac{\mathrm{Number}\;\mathrm{of}\;\mathrm{alarms}}{T-a},$$

where *T* is the length of the testing period. We further plot $\left(R_{\mathrm{Alarm}},R_{\mathrm{Miss}}\right)$ in the error diagram.

7. Steps 4 to 6 are continuously repeated until all the rebounds are successfully predicted.

**Figure 5:**
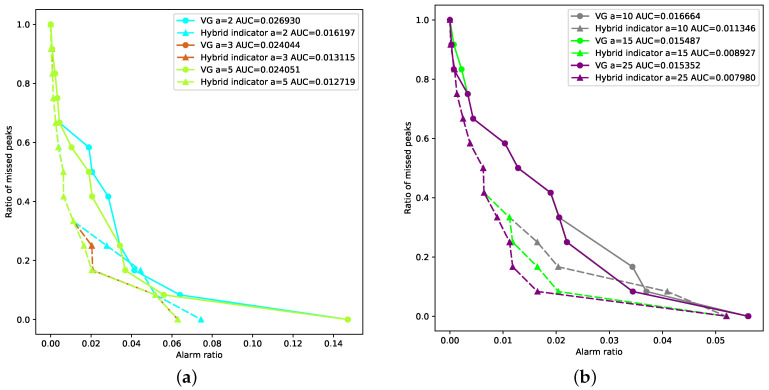
Error diagrams. (**a**) Short-term prediction of VG-based and hybrid indicator. (**b**) Long-term prediction of VG-based and hybrid indicator.

Figure 5 compares the short-term and long-term prediction performance of our proposed method (i.e., the hybrid indicator) with the VG-based indicator. We use the same color to represent the same forward-looking period *a*. Solid lines with circular icons correspond to the VG-based method, while dashed lines with triangular icons represent the results for the hybrid indicator. It can be observed that our proposed hybrid indicator outperforms the benchmark VG-based indicator for any forward-looking period.

Table 2 shows the *p*-values of the predictions for all the forward-looking periods a=2,3,5,10,15,25. It can be observed that, for each forward-looking period *a*, the *p*-value of the VG-based indicator is smaller than 0.03. This indicates that the VG-based indicator is superior to a random guess on a significance level 3%. In other words, the predictability of the VG-based indicator is significant on a significance level 3%, which matches the result in the previous paper [16]. Similarly, our proposed hybrid indicator is superior to a random guess at a 2% significance level. We can conclude that the predictability of our proposed hybrid indicator is significant at a significance level of 2%. We can further observe that the *p*-value of our proposed indicator is smaller than the *p*-value of the VG-based indicator for each *a*, indicating that our proposed method constantly outperforms the benchmark method.

**Figure 6:**
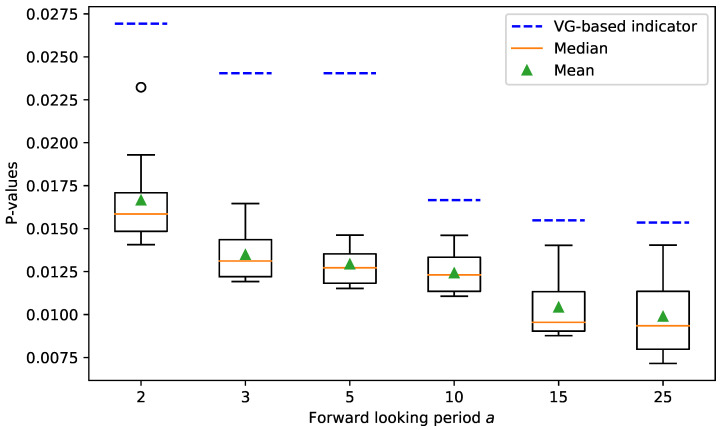
Statistics of the *p*-values for different parameters.

We finally test the robustness of our proposed method. We choose $T_{\mathrm{Alm}}$ from {2,3,⋯,10} and calculate the nine corresponding *p*-values for each forward-looking period *a*. The box plot in Figure 6 shows the statistics of the *p*-values for different $T_{\mathrm{Alm}}$. In this figure, each box represents the nine *p*-values for a forward-looking period *a*. The green dots are the mean, while the orange solid lines are medians. The lower and upper edges of each box are the first and third quartiles, which are denoted as $Q_{1}$ and $Q_{3}$, respectively. The top and bottom boundaries are $Q_{3}+1.5\mathrm{IQR}$ and $Q_{1}-1.5\mathrm{IQR}$, where $\mathrm{IQR}=Q_{3}-Q_{1}$ is the interquartile range. Points out of the upper and lower boundaries are outliers. For the convenience of comparison, we label the *p*-value of VG-based indicator with the blue dashed line for each *a*. It can be observed that the *p*-values of our method are constantly smaller than the VG-based benchmark, indicating that the performance of our proposed framework is consistently better. We can conclude that our proposed framework is robust to the choice of the free parameter $T_{\mathrm{Alm}}$.

## 5. Discussion

The findings of this study indicate that incorporating the analysis of a time-evolving network can improve the performance of traditional time series models. Qualitative observations show the rapid decline of SVD entropy during financial crashes and suggest that the network-based indicator helps with reducing the false alarm rate. Quantitative comparisons further confirm that the predictive power is improved by combining the network-based indicator.

According to the qualitative observations, the SVD entropy of the stock correlation network of the NYSE Composite Index decreases significantly during stock market crashes. This is consistent with the existing studies of various stock indices based on different definitions of network entropy [13,14,15,39]. For example, Zhang et al. [15] have observed decreases in the von Neumann entropy and the graph motif entropy of the NYSE network during financial crashes. Furthermore, it is also observed that the structural entropy declines rapidly during crisis periods in the stock correlation network of FTSE100 and NIKKEI225 [13]. Since “entropy” can be interpreted as “diversity”, such observations suggest that the “structural diversity” of the stock market commonly declines during financial crashes, and the recovery of structural diversity reflects the recovery of the market. We may further interpret this econophysics phenomenon from the perspective of behavioral finance. The structural diversity of the stock market may represent investors’ perceptions in the heterogeneity of different firms. During financial crashes, spillover effects and investors’ herding behavior may erase the differences between “good” and “bad” firms, and thus can eliminate the structural diversity of the stock market.

In addition, it can be observed that the network-based indicator helps with reducing the false alarm rate of the time series-based indicator. This finding can shed light on a better understanding of the stock market crashes. Traditionally, research on stock market crashes focuses on modeling the price time series of stock indices [16,17,40,41,42,43]. In particular, the benchmark indicator of this study (i.e., the VG-based LPPL indicator) [16] captures the faster-than-exponential decline in stock index price. However, our experimental results show that detection and prediction based on the time series alone can contain a number of false alarms. In other words, a rapid decline in stock index prices does not necessarily mean a financial crash. An intuitive explanation is that the plunge of highly weighted sectors or companies may drive down the stock index, but as long as the plunge is not propagated through the stock network, it will not trigger the crash of the whole market. By combining network-based and time series-based indicators, this study contributes to the LPPL-related literature and highlights that anomalous topological changes of the stock correlation network can be important criteria for confirming the occurrence and predicting the recovery of stock market crashes.

Quantitative comparisons confirm that the incorporation of SVD entropy improves the predictive power of the time series-based model. This finding agrees with the existing literature on the predictive power of SVD entropy for stock market dynamics [24,25,26,27]. For a number of representative stock indices such as the Dow Jones Industrial Average [24], the Shenzhen Component Index [25] and the Shanghai Component Index [27], it has been found that the SVD entropy of the constituent stock network has a predictive ability to the dynamics of the stock index. However, the existing literature mainly focuses on examining the predictive power of SVD entropy using the Granger causality test, without developing methods or models to practically leverage such predictive ability. This study contributes to this research gap by proposing a framework that jointly considers the SVD entropy and the stock index price. It is demonstrated that the predictive power of the SVD entropy can be practically applied.

It should be acknowledged that this work has several limitations. First, this study transforms the time-evolving stock correlation network into the time series of a network statistic (i.e., the SVD entropy) and therefore ignores the finer topology in the network. In particular, our proposed framework is not able to recognize the potential micro- and meso-level structural changes. This limitation also hinders a more in-depth observation of the topological changes during and after financial crashes. As a consequence, a number of critical research questions are left unanswered. For example, it is still unclear if similar micro- and meso-level patterns can be observed among multiple financial crashes throughout history. If so, developing models and methods based on such patterns to detect crashes and predict rebounds can be another interesting research topic. Another limitation of this study is that an in-depth analysis of the mechanism is not performed. For example, this study focuses on the diagnosis of major financial crashes. However, we have also observed rapid declines in stock indices accompanied by anomalous network changes during some periods that are not regarded as major financial crashes. Furthermore, the topological changes in the stock correlation network have become increasingly drastic from 1968 to the present. The causes of these phenomena are still unclear.

This study has several practical implications for professionals in a variety of fields. For both institutional and individual investors, our proposed rebound indicator provides a referential signal for market timing strategies. It can be observed that weak signals start to appear when a stock market crash occurs. As the stock market crash progresses, the rebound indicator gradually gets stronger and clusters around certain dates. When the rebound indicator reaches the peak and starts to decline, a change of regime is more likely to occur. Therefore, in the aftermath of stock market crashes, investors can start applying long strategies after observing the clustering and peaking of the rebound indicator. For policymakers, this study provides useful information that can help with the detection and management of market risks. When abnormal changes in the stock network are detected, policymakers can be alerted to conduct further in-depth research to determine whether a systemic risk exists. Furthermore, this study recommends introducing policies to stabilize key firms and sectors once a systemic risk occurs, since the contagion of risks may destroy the market structure and trigger herding behavior of investors. From a broader perspective, this study also has practical implications for machine learning researchers and algorithm developers. Our findings highlight the importance of incorporating stock network analysis into traditional time series models. Novel algorithms can be developed along this direction to better diagnose and forecast extreme financial events.

## 6. Conclusions

This study proposes a framework to diagnose stock market crashes and predict the subsequent price rebounds by jointly modeling plunges in the stock index price and abnormal changes in the stock correlation network. Experiments based on the NYSE Composite Index show that our proposed framework outperforms the benchmark VG-based LPPL model. In line with the existing literature, we observe rapid declines of the stock network’s SVD entropy during market crashes. It suggests that the elimination of structural diversity in the stock market can be an important characteristic of financial crashes. In addition, it is observed that we reduce the false alarm rate of the LPPL-based indicator by incorporating the network anomaly-based indicator. This finding can shed light on bridging the gap between stock network analysis and financial time series modeling, which are often considered as two relatively independent research directions. From the perspective of market participants (e.g., policymakers and investors), this study can provide referential signals for risk management and market timing strategies.

The main limitation of this paper is the conversion of the time-evolving network into a time series, thus losing the micro- and meso-level topological patterns. Future research can benefit from using representation learning methods (e.g., graph neural network and tensor decomposition) that can directly process dynamic networks. Techniques for interpretable machine learning can also be applied to identify specific topological patterns in the course of market crashes and recoveries. Moreover, future research can be conducted to reveal the mechanism of abnormal topological changes in stock correlation networks during market crashes. Finally, practitioners can integrate our proposed rebound indicator into their trading strategies to control risk and make profits in the aftermath of stock market crashes.

## Figures and Tables

**Table 1:** Major stock market crashes in the U.S. from January 1986 to May 2021.

Name	Date
Black Monday	19 October 1987
Friday the 13th mini-crash	13 October 1989
Early 1990s recession	3 July 1990
1997 Asian financial crisis	2 July 1997
Russian financial crisis	17 August 1998
Dot-com bubble	10 March 2000
September 11 attacks	11 September 2001
Stock market downturn of 2002	19 March 2002
Financial crisis of 2007–2008	31 October 2007
2009 Icelandic financial crisis	20 January 2009
European sovereign debt crisis	27 April 2010
August 2011 stock markets fall	1 August 2011
2015–2016 stock market selloff	18 August 2015
2018 cryptocurrency crash	20 September 2018
2020 stock market crash	24 February 2020

**Table 2:** The *p*-values of the predictions for all the forward-looking periods.

Forward-Looking Period *a*	VG-Based Indicator	Our Proposed Hybrid Indicator
2	0.026930	0.016197
3	0.024044	0.013115
5	0.024051	0.012719
10	0.016664	0.011346
15	0.015487	0.008927
25	0.015352	0.007980

**Figure A1:**
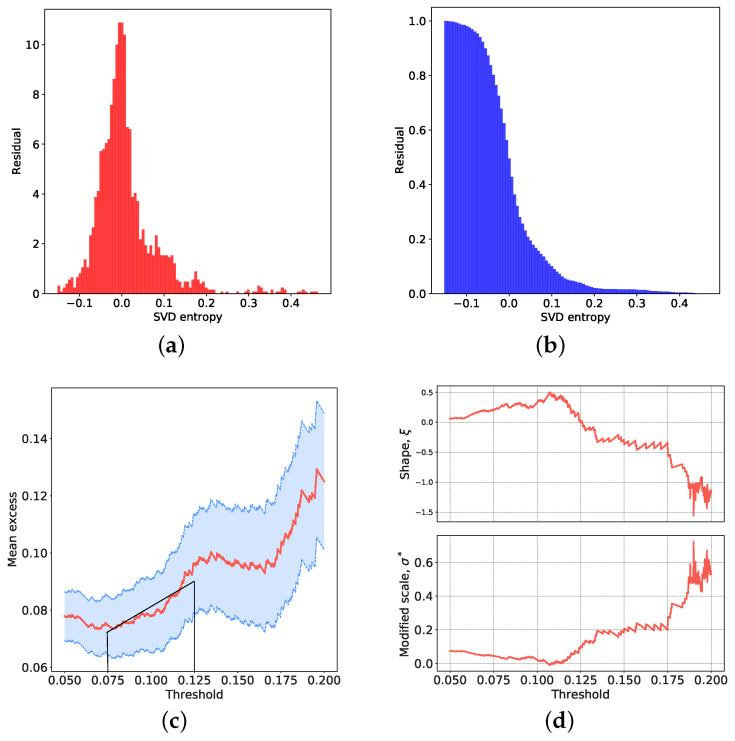
Selecting the threshold for the NYSE Composite Index. (**a**) Empirical PDF. (**b**) Empirical CCDF. (**c**) MRL Plot. (**d**) Parameter Stability Plot.

**Figure A2:**
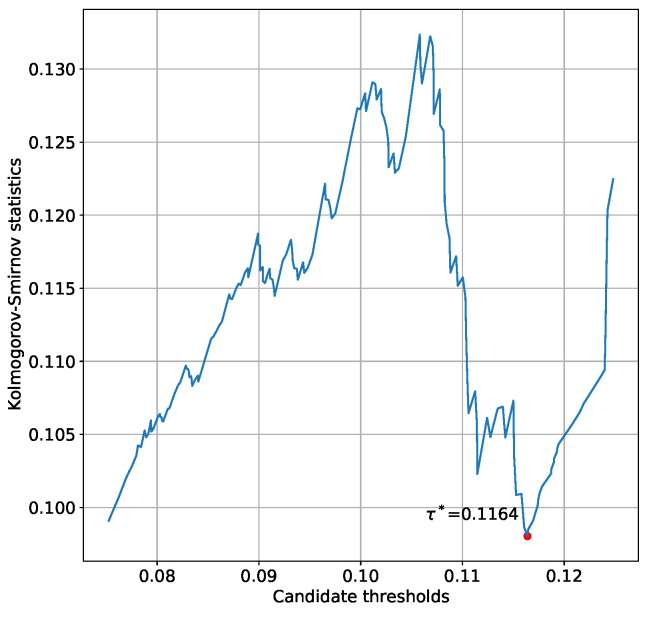
Threshold selection based on the Kolmogorov–Smirnov statistic.

## Data Availability

This study uses publicly available datasets, which can be found in http://finance.yahoo.com and https://en.wikipedia.org/w/index.php?title=List_of_stock_market_crashes_and_bear_markets (accessed on 25 November 2021).

## Appendix A. Trend Labeling Algorithm

For completeness, we illustrate the detailed algorithm for labeling the trend of the stock index price time series, which is proposed by Wu et al. [18]. The detailed procedure is introduced in Algorithm A1. The input of this algorithm is the time series of daily prices $P=\left[p_{1},p_{2},p_{3},\cdots ,p_{T}\right]$, as well as the predetermined proportion threshold w∈(0,1). The output of the algorithm is the corresponding label vector $Y=\left[{\mathrm{label}}_{1},\;{\mathrm{label}}_{2},\;{\mathrm{label}}_{3},\;\ldots ,\;{\mathrm{label}}_{T}\right]$, where

(A1) $${\mathrm{label}}_{t}=\left\{\begin{array}{cc} 1, & \;\mathrm{if}\;a\;\mathrm{rebound}\;\mathrm{occurs}\;\mathrm{on}\;\mathrm{day}\;t.\\ -1, & \;\mathrm{if}\;\mathrm{day}\;t\;\mathrm{is}\;a\;\mathrm{turning}\;\mathrm{point}\;\mathrm{from}\;\mathrm{uptrend}\;\mathrm{to}\;\mathrm{downtrend}.\\ 0, & \;\mathrm{otherwise}\; \end{array}\right.$$

Before executing the labeling algorithm, the local peak and the local trough are initialized as $\mathrm{LP}=p_{1}$ and $\mathrm{LT}=p_{1}$. The trend label is initialized as trend=0, where trend=1 indicates an upward trend and trend=−1 indicates a downward trend. The label vector *Y* is initialized as Y=[0,0,⋯,0]. The index of the first financial extreme *t* is initialized as t=0.

**Algorithm A1** Method for labeling rebounds
**Input:** $P=\left[p_{1},p_{2},p_{3},\cdots ,p_{T}\right]$; w=15 **Output:** $Y=\left[{\mathrm{label}}_{1},\;{\mathrm{label}}_{2},\;{\mathrm{label}}_{3},\ldots ,\;{\mathrm{label}}_{T}\right]$. 1: **for**i=1 to *T* **do** 2: **if** P[i]>LP(1+w) **then** 3: Labeling the first peak: LP=P[i], trend=1, t=i, Y[i]=1. 4: **break** 5: **end if** 6: **if** P[i]<LT(1−w) **then** 7: Labeling the first trough: LT=P[i], trend=−1, t=i, Y[i]=1. 8: **break** 9: **end if** 10: **end for** 11: **for**i=t to *T* **do** 12: **if** trend==1 **then** 13: **if** P[i]>LP **then** 14: Updating the local peak: LP=P[i] 15: **end if** 16: **if** P[i]<LP(1−w) **then** 17: Labeling the turning point: LT=P[i], Y[i]=−1, trend=−1. 18: **end if** 19: **end if** 20: **if** trend==−1 **then** 21: **if** P[i]<LT **then** 22: Updating the local trough: LT=P[i] 23: **end if** 24: **if** P[i]>LT(1+w) **then** 25: Labeling the turning point: LP=P[i], Y[i]=1, trend=1 26: **end if** 27: **end if** 28: **end for** 29: **return***Y*;

## Appendix B. Algorithm for the Generation of a Training Set

This appendix describes the algorithm for the generation of training set, whose detailed procedure is described in Algorithm A2. The input of this algorithm is the first *N* data points of the indicator time series $I=\left[I_{1},I_{2},\cdots ,I_{N}\right]$ with labeled financial crashes $L=\left[l_{1},l_{2},,\cdots ,l_{T}\right]$, where

(A2) $$l_{t}=\left\{\begin{array}{cc} 1, & \;\mathrm{if}\;\mathrm{day}\;t\;\mathrm{is}\;\mathrm{during}\;a\;\mathrm{financial}\;\mathrm{crash}.\\ 0, & \;\mathrm{otherwise}\; \end{array}\right.$$

**Algorithm A2** Generating training set
**Input:** First *N* indicators $I=\left[I_{1},I_{2},\cdots ,I_{N}\right]$; Corresponding event labels $L=\left[l_{1},l_{2},,\cdots ,l_{N}\right]$; The sliding window length *d*. **Output:** Training input set $S_{x}$ and target output set $S_{y}$. 1: i,j=0, W←[] 2: **while** i<N**do** 3: **while** j<N−1 and $l_{j}==l_{j+1}$ **do** 4: j=j+1 5: **if** $l_{i}==0$ **then** 6: $W_{i}=I\left[i:j\right]$, $W\leftarrow W\cup W_{i}$ 7: **end if** 8: j=j+1, i=j 9: **end while** 10: **end while** 11: $S_{x},S_{y}\leftarrow \left[\right]$ 12: **for** i=1 to length(W) **do** 13: **for** j=d+1 to length($W_{i}$) - 1 **do** 14: $S_{x}\leftarrow S_{x}\cup W_{i}\left[j-d:j\right]$ 15: $S_{y}\leftarrow S_{y}\cup W_{i}\left[j+1\right]$ 16: **end for** 17: **end for** 18: **return** $S_{x},S_{y}$

## Appendix C. Obtaining Optimal Threshold Based on Extreme Value Theory

In this appendix, we describe in detail the method of obtaining the optimal threshold for residuals, which is used to distinguish normal residuals from abnormal residuals. The training data that we are using are the SVD entropy of the NYSE financial network from 10 February 1986 to 3 January 1994. The residuals are calculated as

(A3) $$X_{t}=F\left(I_{t-d},\cdots ,I_{t-1}\right)-I\left(t\right).$$

In this case, we use the Prophet forecasting model [34] as the predictor F(·), whose open-source implementation is available at https://github.com/facebook/prophet (accessed on 25 November 2021).

The general procedure for obtaining τ is summarized in Algorithm A3. Such algorithm mainly contains three key steps: (1) generating possible candidates, (2) estimating parameters for GPD; (3) selecting the optimal threshold based on the Kolmogorov–Smirnov statistic. The detailed procedure is described as follows.

### Appendix C.1. Generating Possible Candidates

The possible candidates for the threshold are selected based on a hybrid approach combining EVT and observation of the data. The two most common methods for selecting thresholds in EVT applications are the mean residual life (MRL) plot and the parameter stability plot [44,45]. The MRL plot shows the relationship between the *mean excess* (i.e., the average of $Y_{\tau }$) and τ. Its theoretical foundation is the conclusion proved by [46], that is, if the threshold $\tau_{0}$ enables $X-\tau_{0}$ to be the GPD excess, for any higher threshold $\tau >\tau_{0}$, the average value of X−τ (i.e., the *mean excess*) is E(X−τ∣X>τ)=(σ+ξτ)/(1−ξ), which is linear to τ. Therefore, the MRL plot is approximately linear within a suitable range of τ. Meanwhile, too low a threshold τ would violate the assumption of extreme value theory and thus lead to a tail distribution that does not obey the GPD. Too high a threshold will lead to too few excesses, which brings a large uncertainty to the parameter estimation. Therefore, the MRL plot is not a straight line for a too high or too low threshold τ.

**Algorithm A3** Finding the threshold τ based on EVT
**Input:** The residuals for the first *N* days $X=\left[X_{1},X_{2},X_{3},\cdots ,X_{N}\right]$. **Output:** $\tau^{*}$, $\xi (\tau^{*})$, and $\sigma (\tau^{*})$. 1: Generating possible candidates for the threshold $\tau =\left[\tau_{1},\tau_{2},\tau_{3},\cdots \right]$. 2: **for** τ in τ **do** 3: $Y_{\tau }\leftarrow \left\{X_{i}-\tau |X_{i}>\tau \right\}$; 4: Estimating parameters $\hat{\xi }\left(\tau \right)$ and $\hat{\sigma }\left(\tau \right)$ based on $Y_{\tau }$; 5: Calculating the Kolmogorov–Smirnov statistic $\mathrm{KS}\left(\tau \right)$ between $\mathrm{GPD}\left(\hat{\xi }\left(\tau \right),\hat{\sigma }\left(\tau \right)\right)$ and $Y_{\tau }$; 6: **end for** 7: **return** $\tau^{*}$ with the smallest $\mathrm{KS}\left(\tau \right)$, as well as two parameters $\xi (\tau^{*})$ and $\sigma (\tau^{*})$ of the GPD.

Generally, the MRL plot can provide a rough range of the proper threshold. However, the MRL plot in practical applications may not be ideally straight. In fact, it is widely recognized by researchers that the interpretation of practical MRL plots can be challenging [33]. As a supplementary method, the parameter stability plot involves plotting the estimated parameters $\hat{\sigma }\left(\tau \right)$ and $\hat{\xi }\left(\tau \right)$ of the GPD model against τ. Within the proper range of τ, $\hat{\sigma }\left(\tau \right)$ and $\hat{\xi }\left(\tau \right)$ should be near-constant, so the parameter stability plot should be an approximately horizontal straight line.

In addition, observing the probability density function (PDF) and complementary cumulative distribution function (CCDF) of the data also gives us an intuitive understanding of the appropriate threshold values. Therefore, we plotted these four figures (i.e., PDF, CCDF, the MRL plot and the parameter stability plot) in Figure A1.

In this figure, (a), (b), (c) and (d) are the PDF, CCDF, the MRL plot and the parameter stability plot of the residual. From the PDF, it can be observed that the major part of this distribution approximates a normal distribution, which is consistent with our assumptions on the residual ϵ(t). Furthermore, the distribution has a heavy tail where the outliers are located. The CCDF is also obviously heavy-tailed, which supports our hypothesis based on EVT. Combining the approximately linear part of the MRL plot and the near-constant part of the parameter stability plot, we determined the search interval for the threshold τ as (0.075,0.125). It is worth noting that we actually relax the search interval. The possible candidates for the threshold are the data points that lie within the search interval, that is, $T=\left\{X_{t}|0.075<X_{t}<0.125\right\}$.

### Appendix C.2. Estimating Parameters for GPD

For each possible candidate τ∈T, we assume that the threshold excesses $Y_{\tau }=\left\{X_{i}-\tau |X_{i}>\tau \right\}$ follow the GPD. The parameters $\hat{\xi }\left(\tau \right)$ and $\hat{\sigma }\left(\tau \right)$ are estimated based on the maximum likelihood estimation (MLE), which is considered as a more efficient and robust approach compared with the method of moments (MOM) method and the probability weighted moments (PWM) method [47].

We follow the parameter estimation procedure illustrated by [29], which is based on Grimshaw’s trick [48]. The MLE method aims at maximizing the log-likelihood function of GPD:

(A4) $$\ell \left(\xi ,\sigma \right)=\log L\left(\xi ,\sigma \right)=-N_{t}\log\sigma -\left(1+\frac{1}{\xi }\right)\sum_{i=1}^{N_{t}}\log\left(1+\frac{\xi }{\sigma }Y_{i}\right)$$

where $Y_{i}\in Y_{\tau }$ are the threshold excesses. The global optimal solutions $\xi^{*}$ and $\sigma^{*}$ that maximize ℓ(ξ,σ) must satisfy

(A5) $$\nabla \ell (\xi^{*},\sigma^{*})=0.$$

Grimshaw [48] has demonstrated that the solutions of Equation (A5) satisfy the following conditions

(A6) $$\left\{\begin{array}{c} u(x)v(x)=1\;\\ u\left(x\right)=\frac{1}{N_{t}}\sum_{i=1}^{N_{t}}\frac{1}{1+xY_{i}}\;\\ v\left(x\right)=1+\frac{1}{N_{t}}\sum_{i=1}^{N_{t}}\log\left(1+xY_{i}\right) \end{array}\right.,$$

where $x=\xi^{*}/\sigma^{*}$. Solutions of Equation (A6) can be obtained by performing numerical root searches in the interval $\left(x_{\min},x_{\max}\right)$. The lower bound of the interval is $x_{\min}=-1/\max\left\{Y_{\tau }\right\}$ and the upper bound is

(A7) $$x_{\max}=2\frac{{\bar{Y}}_{\tau }-\min\left\{Y_{\tau }\right\}}{{\left(\min\left\{Y_{\tau }\right\}\right)}^{2}},$$

where ${\bar{Y}}_{\tau }$ is the mean of $Y_{\tau }$. For each solution *x* of Equation (A6), the corresponding ξ and σ can be calculated as,

(A8) $$\left\{\begin{array}{c} \xi =v\left(x\right)-1\;\\ \sigma =\xi /x \end{array}\right.$$

For all solutions of Equation (A6), we compute their corresponding ξ and σ, which are substituted into Equation (A4) to compute ℓ(ξ,σ). We select the ξ and σ that maximize ℓ(ξ,σ) as the estimated parameters of the GPD.

### Appendix C.3. Threshold Selection Based on the Kolmogorov–Smirnov Statistic

In the previous step, we estimated the parameters $\hat{\xi }\left(\tau \right)$ and $\hat{\sigma }\left(\tau \right)$ of GPD for each possible threshold τ∈T. Here we calculate the Kolmogorov–Smirnov (KS) statistic between the empirical distribution function (EDF) of $Y_{\tau }$ and the cumulative distribution function (CDF) of GPD with parameters $\hat{\xi }\left(\tau \right)$ and $\hat{\sigma }\left(\tau \right)$. Figure A2 plots the KS statistics against the threshold τ for the residual. We choose the optimal threshold $\tau^{*}$ such that the corresponding KS statistic is minimized, which means that EDF and CDF are the closest and the best fit is obtained. As is shown in Figure A2, the optimal threshold is $\tau^{*}=0.1164$, with $\xi (\tau^{*})=0.3004$ and $\sigma (\tau^{*})=0.0276$.

## Appendix D. Execution Step

This appendix presents the detailed procedure of the execution step. With the input of streaming data, we keep updating the training set, which is used to continuously fine-tune the predictor. It is worth noting that we distinguish between “normal” and “abnormal” data based on EVT, and only include “normal” data into the updated training set. In this way, the predictor is capable of tracking the "normal" changes in the dynamics of the time series.

**Algorithm A4** Execution step
1: The set for new training samples W←[]. 2: c=0 3: **for** t>N**do** 4: ${\hat{I}}_{t}=F\left(I_{t-d},\cdots ,I_{t-1}\right)$ 5: $X_{t}={\hat{I}}_{t}-I_{t}$ 6: **if** $X_{t}>\tau$ **then** 7: Raise an alarm with the alarm index $F_{\xi ,\sigma }\left(X_{t}-\tau \right)$. 8: Empty new training sample set: W←[] 9: **else** 10: Append new training sample set: $W\leftarrow W\cup I_{t}$. 11: **if** length(W)>d **then** 12: $S_{x}\leftarrow S_{x}\left[1:\;\right]\cup W\left[1:d\right]$ 13: $S_{y}\leftarrow S_{y}\left[1:\;\right]\cup W\left[d+1\right]$ 14: W←[] 15: c←c+1 16: **end if** 17: **end if** 18: **if** mod(c,K)==0 **then** 19: Train the predictor F(·) by the updated training set $S_{x}$ and $S_{y}$. 20: **end if** 21: **end for**
